# The Association Between Concentrations of Arginine, Ornithine, Citrulline and Major Depressive Disorder: A Meta-Analysis

**DOI:** 10.3389/fpsyt.2021.686973

**Published:** 2021-11-18

**Authors:** Mingyue Fan, Xiao Gao, Li Li, Zhongyu Ren, Leanna M. W. Lui, Roger S. McIntyre, Kayla M. Teopiz, Peng Deng, Bing Cao

**Affiliations:** ^1^Department of Public Health and Management, Chongqing Three Gorges Medical College, Chongqing, China; ^2^Key Laboratory of Cognition and Personality, Faculty of Psychology, Ministry of Education, Southwest University, Chongqing, China; ^3^National Demonstration Center for Experimental Psychology Education, Southwest University, Chongqing, China; ^4^College of Physical Education, Southwest University, Chongqing, China; ^5^Mood Disorders Psychopharmacology Unit, Toronto, ON, Canada; ^6^Yubei Center for Disease Control and Prevention, Chongqing, China

**Keywords:** arginine, depression, nitric oxide, metabolism, catabolism, bipolar disorder, cognition

## Abstract

Alterations in the peripheral (e.g., serum, plasma, platelet) concentrations of arginine and its related catabolic products (i.e., ornithine, citrulline) in the urea and nitric oxide cycles have been reported to be associated with major depressive disorder (MDD). The meta-analysis herein aimed to explore the association between the concentration of peripheral arginine, its catabolic products and MDD, as well as to discuss the possible role of arginine catabolism in the onset and progression of MDD. PubMed, EMBASE, PsycINFO and Web of Science were searched from inception to June 2020. The protocol for the meta-analysis herein has been registered at the Open Science Framework [https://doi.org/10.17605/osf.io/7fn59]. In total, 745 (47.5%) subjects with MDD and 823 (52.5%) healthy controls (HCs) from 13 articles with 16 studies were included. Fifteen of the included studies assessed concentrations of peripheral arginine, eight assessed concentrations of ornithine, and six assessed concentrations of citrulline. Results indicated that: (1) the concentrations of arginine, ornithine, and citrulline were not significantly different between individuals with MDD and HCs when serum, plasma and platelet are analyzed together, (2) in the subgroups of serum samples, the concentrations of arginine were lower in individuals with MDD than HCs, and (3) concurrent administration of psychotropic medications may be a confounding variable affecting the concentrations of arginine, ornithine, and citrulline. Our findings herein do not support the hypothesis that arginine catabolism between individuals with MDD and HCs are significantly different. The medication status and sample types should be considered as a key future research avenue for assessing arginine catabolism in MDD.

## Introduction

Major depressive disorder (MDD) is one of the most common mental disorders affecting more than 350 million people worldwide (1, 2). Furthermore, MDD is one of the leading causes of global burden of disease. Major depressive disorder affects approximately 16% of the world's population (3), continues to be a major cause of disability worldwide, and is the number one cause of suicide (4). Excessive inflammation and neurodegenerative changes have been reported in MDD, both of which have been associated with cognitive impairment (5). However, the underlying pathology of MDD remains poorly understood, in part due to the heterogeneity of genetic and environmental factors related to proposed mechanisms of action (6). Moreover, the pathogenesis of acute depression may be different from recurrent or chronic depression, which is characterized by long-term decline in social or occupational function and cognitive ability (7).

Intensified research efforts are devoted to predicting onset of MDD and treatment response through exploring possible biomarkers/biosignatures including but not limited to metabolic pathways (8–10). Extensive literature indicates that amino acids may be candidate biomarkers for a variety of diseases, including metabolic syndrome, cancer, and mental disorders (11–16). With increasing research focusing on the dysfunction of oxidative and nitrosative stress in MDD, arginine catabolism regulation has received increasing attention (17–19).

Arginine is a semi-essential amino acid and a substrate of important metabolic pathways in the physiological processes of the central nervous system and immune defense [e.g., urea and nitric oxide (NO) cycles] (20, 21). Arginine is transformed into NO and citrulline by endothelial nitric oxide synthase (eNOS), inducible nitric oxide synthase (iNOS) and neuronal nitric oxide synthase (22, 23). The alteration of arginine may lead to abnormalities of NO metabolism and the urea cycle metabolic pathway (24, 25).

The dysregulation of the L-arginine-NO metabolic pathway has been linked to the pathogenesis of severe depression (26, 27). For example, a study reported a positive correlation between increased plasma NO concentrations and suicide attempts in individuals with mild depression (28). A separate study reported that the inhibition of NOS induced antidepressant effects in rats (29). Hitherto, reducing or blocking the synthesis of NO (i.e., blocking NOS) in the brain may be protective against depression (i.e., antidepressant effects) (29, 63).

Available clinical evidence has demonstrated that the NO signaling pathway is associated with schizophrenia, anxiety disorders, and affective disorders (30). Notably, the NO system has previously been reported to be a potential target of antidepressant and anti-anxiety drugs in acute therapy and prevention (31).

Previous cross-sectional studies among patients with MDD and experimental studies based on animal models of depression have reported the altered arginine levels in blood related to the catabolite and NO imbalance in pathophysiology of MDD (18, 32). The dysfunction of NO signaling pathway has been suggested as a nexus between MDD and commonly encountered comorbidities via platelet activation, endothelial dysfunction (i.e., low circulating endothelial NO concentrations and impaired vasodilation), and elevated concentrations of proinflammatory circulating cytokines (33, 34).

Arginine, citrulline, and ornithine are key amino acids of the urea cycle (35). Arginine is the substrate for arginase, the enzyme that produces urea while converting arginine to ornithine (36). Previous studies have shown that arginase activity is elevated in people with depression (37). Moreover, L-arginine is reported to be a risk factor for the development of mild depression (38). It has been separately reported that patients with depression have lower circulating L-arginine concentrations (33). Arginine has also been reported to affect concentrations of aminobutyric acid and glutamate in the prefrontal cortex of the brain, which are important to cellular bioenergetics and oxidative stress (39). In addition, two clinical trials have shown that ketamine and esketamine, glutamatergic N-methyl-D aspartate receptor (NMDAR) antagonists with established antidepressant effects, contributed to the changes in arginine in the urea cycle (40, 41). Taken together, extensive literature has supported the notion that arginine may be implicated in mechanisms that are relevant to MDD.

Ornithine is a metabolite of arginine (42). Citrulline is derived not only from the production of NO but also from the action of the enzyme ornithine carbamoyltransferase (43). Previous findings regarding the function of arginine catabolism underlying MDD are inconsistent. L-Arginine competes with asymmetric dimethylarginine for NOS (44) and increases endothelial NO production and reverses the endothelial dysfunction associated with vascular risk factors (64). L-citrulline can be converted to L-arginine via the citrulline-NO cycles as well as the urea cycle. The possibility that decreased L-cirtulline contributes to decreased L-arginine concentrations in physically healthy patients with MDD cannot be excluded (45). It has been previously demonstrated that L-arginine may have the potential to treat MDD (46). Taken together, the foregoing studies provide the rational to assess the potential association between L-arginine concentrations and MDD.

To our knowledge, there has been no previous meta-analysis evaluating the association between arginine catabolism and MDD. Herein, the current meta-analysis aims to compare peripheral arginine and its related catabolic products (i.e., ornithine and citrulline) between patients with MDD and healthy controls (HCs) (i.e., healthy volunteers who do not have current or previous history of mental illness).

## Methods

### Literature Search

**Figure 1:**
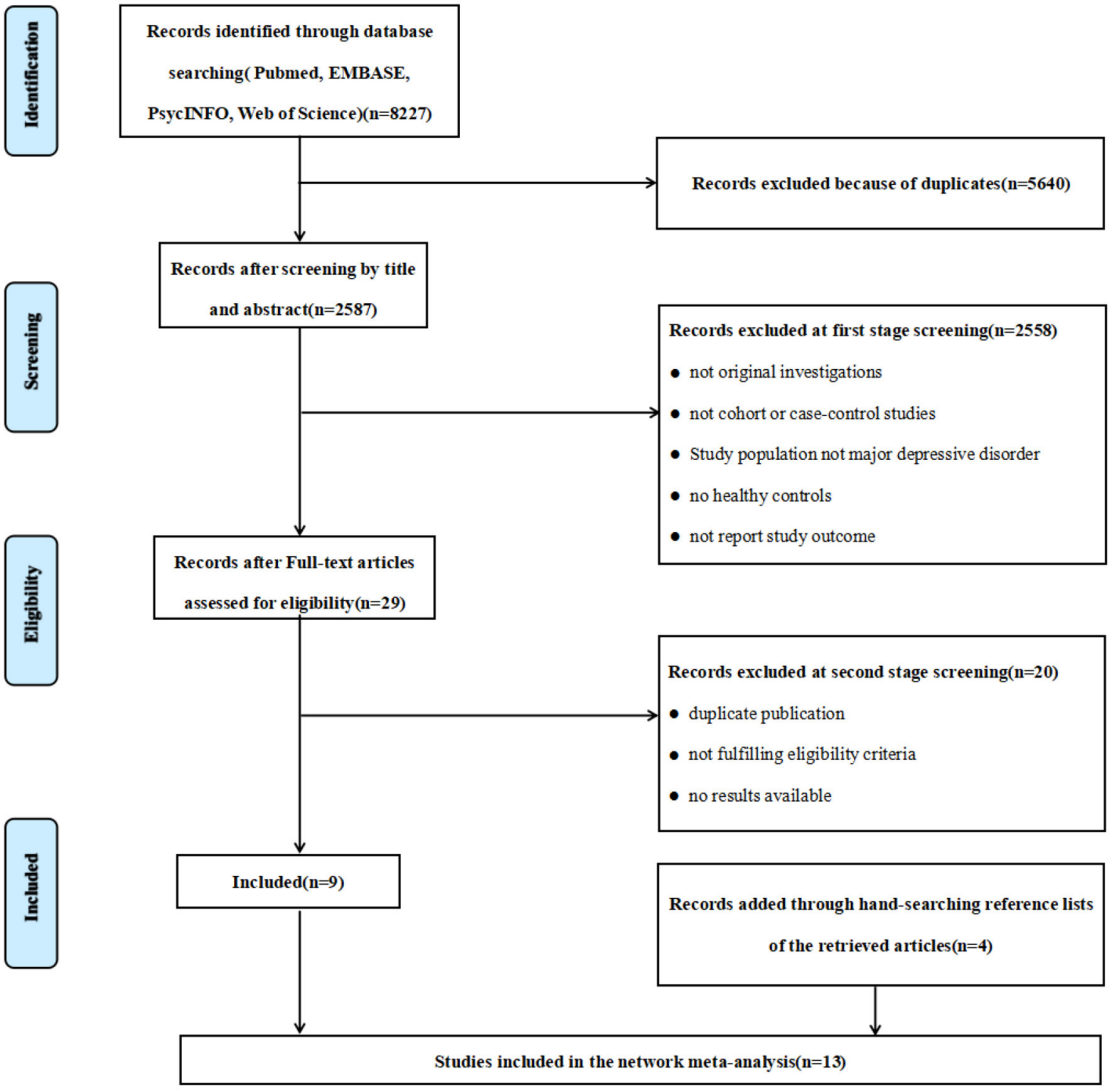
PRISMA flow diagram of study selection process.

We performed this study according to the Preferred Reporting Items for Systematic Reviews and Meta-Analyses (PRISMA) (47), and a systematic retrieval of literature from inception to June 2020. The literature search was conducted using the following online databases: PubMed, EMBASE, PsycINFO and Web of Science. The keywords of the search strategy were “major depressive disorder (MDD)”, “depression”, “mood disorder”, “arginine”, “ornithine”, “citrulline”, “L-arginine”, “amino acid”, “argininosuccinate”. The flow diagram outlining the study selection process is shown in Figure 1. The protocol of current meta-analysis has been registered at the Open Science Framework [https://doi.org/10.17605/osf.io/7fn59 (48)].

### Selection Criteria

The inclusion of studies were based on the following criteria: (1) adults (≥ 18 years old) with Diagnostic and Statistical Manual of Mental Disorders (DSM) diagnosed MDD (i.e., DSM-III-R, DSM-IV, DSM-IV-TR and DSM-V); (2) healthy volunteers who are not diagnosed with psychiatric illness and do not have a history of mental illness were used as the HC group; (3) measures of the concentrations of arginine, citrulline, or ornithine (one of which was sufficient) assessed in all subjects; and (4) the study type was either a case-control study or cohort study.

Studies were excluded based on the following criteria: (1) non-original research, articles or conference abstracts; (2) case reports, case studies, case series studies, clinical trials and other articles that did not meet the required research type; (3) non-human studies, the research objective did not include patients with MDD, or the case group contains other mental disorders (e.g., schizophrenia, bipolar disorder); (4) comparison of patients before and after treatment; people with other diseases as control group; (5) concentrations of arginine, citrulline, or ornithine were not available; (6) no full-text or studies were repetitive publications from the same datasets by the same or different authors.

### Data Extraction and Quality Assessment

Two investigators (CB and LL) independently screened and reviewed articles, Supplementary Materials, and extracted relevant information. Several articles may not be captured by our search because their keywords are not exactly matched our search strategies. All reference lists of the retrieved articles were reviewed to identify potential studies for inclusion. The additional articles were manually retrieved from the official website of the journals from the reference lists. The study used standardized tables to extract information for each eligible article. The following information was extracted from each study: first author, publication year, study design, country, geographic location, age, sex (i.e., female, male), body mass index (BMI), type of blood sample specimen required for test (i.e., plasma, serum, platelet), sample detection method [i.e., High Performance Liquid Chromatography (HPLC), Liquid Chromatography Mass Spectrometry (LC-MS), or other], sample size, subjects' mean arginine or ornithine or citrulline concentrations, and standard deviation (SD).

### Statistical Analysis

All data analysis was performed using Stata (version 15.0, Stata Corp LP, College Station, TX, USA). Forest plots was used to estimate the association between arginine, its related catabolic products, and MDD, which was evaluated by standardized mean difference (SMD) with a 95% confidence interval (CI). We assigned weights (%) based on the inverse of the variance. The greater weights represent the greater impact on the combined results. The heterogeneity of all studies was assessed by chi-square statistics and the I-Squared (*I*^2^) test. If *P* < 0.10 or *I*^2^ > 50%, we considered that the heterogeneity had statistical differences and a random effects model would be used. Otherwise, the fixed effect meta-analysis would be applied (9).

Subgroup analysis was performed to explore the potential impact of the inclusion characteristics of the studies on the pooled effect size. The effect sizes of arginine, ornithine and citrulline concentrations were calculated for each subgroup. The subgroups were created based on medication status (prescribed medication vs. medication-free), sample types (plasma, serum, or platelet), published year (before vs. after 2010), regional distribution (Asia, Europe, America, or Oceania) of arginine and detection method (Amino acid Analyzer, HPLC, LC-MS). To create subgroups based on living standards of past decade or earlier, studies were divided into two groups according to their publication date (i.e., before 2010 vs. after 2010) (49, 50). Meta-regression was used to investigate the source of heterogeneity, and the effects of both continuous and categorical factors on the study were assessed simultaneously. Sensitivity analysis was used to investigate whether any single study would have an effect on the heterogeneity of total measurements in each meta-analysis. The funnel plot with Begg's test and Egger's test were used to test publication bias.

The Newcastle-Ottawa Scale was used to detect the risk of bias in observational studies. According to the total scores of the Newcastle-Ottawa Scale, the observational studies were divided into three categories: extremely high risk of bias (0–3 points), high risk of bias (4–6 points) and low risk of bias (7–9 points) (51). The bias risk assessment of the included articles is shown in Supplementary Table 1.

## Results

### Basic Characteristics of Included Studies

A total of 8,227 articles were identified from the preliminary search. After screening titles and abstracts, excluding review articles and duplicated articles, and studies that did not meet the inclusion criteria, 29 articles were selected for full-text review. After evaluation, we identified 9 articles that met the inclusion criteria and were selected for analysis. Four articles were retrieved by manual search. The current meta-analysis includes 13 articles with 16 studies (18, 22, 33, 41, 52–60) (Figure 1). Of the thirteen articles included, ten articles reported the results of a single study each, and 3 articles each of which reported results from two studies.

The basic characteristics of included studies are illustrated in Table 1. The current meta-analysis included 745 (47.5%) individuals with MDD and 823 (52.5%) HCs. 15 of the studies assessed concentrations of arginine, 8 assessed ornithine concentrations, and 6 assessed citrulline concentrations. For the geographic location, 5 studies were conducted in Asia, 6 studies were conducted in Europe, 4 studies were conducted in the United States, and 1 study was conducted in Oceania. Most studies used the detection methods of HPLC (*n* = 10), followed by LC-MS (*n* = 4), and Amino Acid Analyses (*n* = 2). Only one study conducted semi-structured interviews, and the remaining studies conducted structured interviews for diagnosis. Participants in 9 studies had received antidepressant medications, and participants in 7 studies reported to be medication-free. The sample types for 11 studies was plasma, 3 studies were serum, and two were platelets. According to the results of the Newcastle-Ottawa Scale, there were 2 articles defined as “high bias risk” with scores of 6, while the others were all defined as “low bias risk” with scores higher than 7.

**Table 1:** Characteristics of the included studies in the meta-analysis.

**References**	**Country**	**Case**		**Control**		**Sample type**	**Detection method**	**semi-structured interview**	**Diagnosed criteria**	**Disease state and illness duration**	**Arginine**	**Ornithine**	**Citrulline**	Quality Score^*^
		**Sex (M/F)**	**Age (years)**	**Sex (M/F)**	**Age (years)**									
Abou-Saleh MT et al. (52)	Arab	33/30	36.92 ± 11.33	32/38	35.53 ± 10.12	Plasma	Amino Acid Analyser	No	DSM-III-R	All patients were receiving antidepressant medication	Female (MDD:89.15 ± 30.49 μm/L; HCs:65.40 ± 24.13 μm/L)	Male (MDD:69.23 ± 22.6 μm/L HCs:84.69 ± 25.02 μm/L); Female (MDD:65.45 ± 24.03 μm/L HCs:94.89 ± 40.11 μm/L)		6
Maes et al., (53)	Belgium	17/18	49.65 ± 14.78	10/5	47.5 ± 15.0	Serum	HPLC	Yes	DSM-III-R	8 Non-TRD and 27 TRD	Non-TRD:132 ± 27 μmol/l; TRD:121 ± 20 μmol/ml; HCs:137 ± 28 μmol/l			7
Mauri et al., (54)^a^	Italy	15/14	47.41 ± 10.85	12/16	42.46 ± 14.19	Plasma	HPLC	No	DSM-IV	outpatients, without melancholia, drug-free for at least 4 weeks	MDD:83.49 ± 100.59 nmol/ml HCs:87.58 ± 30.92 nmol/ml			7
Mauri et al., (55)^b^	Italy	NA	47.41 ± 10.85	NA	42.46 ± 14.19	Platelet	HPLC	No	DSM-IV	outpatients, without melancholia, drug-free for at least 4 weeks	MDD:0.86 ± 1.99 μmol/10^10^ HCs:1.53 ± 2.94 μmol/10^10^			7
Mitani et al., (56)	Japan	11/12	32.78 ± 12.41	17/14	41.46 ± 19.26	Plasma	HPLC	No	DSM-IV	Six depressed patients were drug-free. Seventeen of 23 depressed patients were on antidepressant medication at the time of examination with dosages ranging from 0 to 225 mg imipramine equivalents	Depression:116.8 (54.8) nnol/ml; HCs: 93.2 (42.8) nnol/ml;			6
Mauri et al., (55)^a^	Italy	5/11	50.18 ± 11.55	9/2	39.90 ± 13.39	Plasma	HPLC	No	DSM-IV	outpatients, all affected by recurrent unipolar depression	MDD: 119.24 ± 129.21 nnmol/ml; HCs: 76.97 ± 32.64 nnmol/ml;			7
Mauri et al., (55)^b^	Italy	NA	50.18 ± 11.55	NA	39.90 ± 13.39	Platelet	HPLC	No	DSM-IV	outpatients, all affected by recurrent unipolar depression	MDD:0.93 ± 1.89 μmol/10^10^; HCs:1.78 ± 2.38 μmol/10^10^			7
Pinto et al., (33)	Brazil	NA	34 ± 4	NA	34 ± 3	Plasma	HPLC	No	DSM-IV	current unipolar major depressive episode or with a diagnosis of comorbid anxiety disorder but no other Axis I disorder	HCs:130 ± 8 μM/ml MDD:104 ± 4 μM/ml	HCs:62 ± 12 μM/ml MDD:97 ± 2 μM/ml		7
Woo et al., (57)	Korea	16/52	65	4/18	68	Plasma	LC-MS/MS	No	DSM-IV	single episode or recurrent	HCs:58.7(39.1–75.2) μmol/l MDD: 69.7(49.0–94.1) μmol/l	HCs:109(88.2–132) μmol/l; MDD: 98.1 (70.5–129) μmol/l	HCs:30.3 (25.7–36.6) μmol/l; MDD: 34.0(25.2–45.6) μmol/l	7
Baranyi et al., (22)	Austria	48/23	49.65 ± 9.84	31/17	46.23 ± 27.51	Plasma	HPLC	No	DSM-IV	all in-patients with major depression	HCs: 97.440(86.117-104.697) μmol/L; MDD: 101.500(86.190- 112.940) μmol/L	HCs: 94.050(72.550-103.775) μmol/L; MDD: 82.64(70.390- 101.290) μmol/L	HCs: 30.648 ± 7.63μmol/L; MDD: 29.887 ± 6.23μmol/L	8
Hess et al., (58)	Canada	20/15	27.06 ± 9.43	20/16	25.97 ± 8.47	Serum	Amino Acid Analyser	No	DSM-IV-TR	current unipolar major depressive episode or with comorbid anxiety disorders	MDD:73.54 ± 21.53 μmol/L HCs: 84.89 ± 25.16 μmol/L		MDD:31.58 ± 6.05 μmol/L; HCs 35.19 ± 6.85 μmol/L	7
Ali-Sisto et al., (18)	Finland	43/56	39.41 ±11.94	124/ 129	55.28 ± 10.08	Serum	LC-MS/MS	No	DSM-IV	outpatients, 84 of the patients used antidepressant medication and 48 used antipsychotic medication	MDD:99.15 (83.1– 115.92) μmol/L; HCs:116.9 (94.87–142.18) μmol/L	MDD:86.43 (69.46–115.93) μmol/L; HCs:86.97 (65.14–107.94) μmol/L	MDD:29.01 (22.01–33.69) μmol/L; HCs:29.44 (23.31–36.61) μmol/L	8
Ogawa et al., (59)^a^	Japan	85/79	41.77 ± 11.89	100/ 117	41.2 ± 13.9	Plasma	HPLC	No	DSM-IV	currently depressed [dMDD] and remitted [rMDD])	dMDD:77.91 ± 21.89μM; HCs:79.20 ± 25.70μM	dMDD:74.86 ± 27.37μM; HCs: 87.09 ± 28.58μM		9
Ogawa et al., (59)^b^	Japan	39/26	43.98 ± 13.10	20/45	43.4 ± 13.4	Plasma	LC-MS	No	DSM-IV	currently depressed [dMDD] and remitted [rMDD])	rMDD:83.73 ± 24.08μM; HCs:90.56 ± 20.67μM			9
Moaddel et al., (41)	America	NA	NA	NA	NA	Plasma	LC-MS/MS	No	DSM-IV	recurrent MDD without psychotic features			HCs: 23.80 ± 7.22μM; MDD:19.25 ± 5.17μM	7
Ozden et al., (60)	America	28/49	41.01 ± 12.22	7/20	38.44 ± 12.23	Plasma	HPLC	No	DSM-IV	first or recurrent episode	MDD:6.64 (7.24) μM; HCs:1.42 (1.32) μM	MDD:7.83 (4.33) μM; HCs:4.51 (1.88) μM	MDD:5.25 (1.85) μM; HCs: 4.42 (1.03) μM	8

*DSM, Diagnostic and Statistical Manual of Mental Disorders; MDD, major depressive disorder; HC, healthy control; HPLC, High Performance Liquid Chromatography; LC-MS, Liquid Chromatography Mass Spectrometry; TRD, treatment-resistant depression; NA, Not Available. Mauri et al., (54)^a^: sample type was plasma; Mauri et al., (54)^b^: sample type was platelet; Mauri et al., (55)^a^: sample type was plasma; Mauri et al., (55)^b^: sample type was platelet; Ogawa et al., (59)^a^: dMDD, depressed (non-remitted) patients with major depressive disorder; Ogawa et al., (59)^b^: rMDD, remitted patients*;

* *The quality score was evaluated by the Cochrane's Newcastle–Ottawa scale*.

### Homogeneity Analysis and Effect Estimation

**Figure 2:**
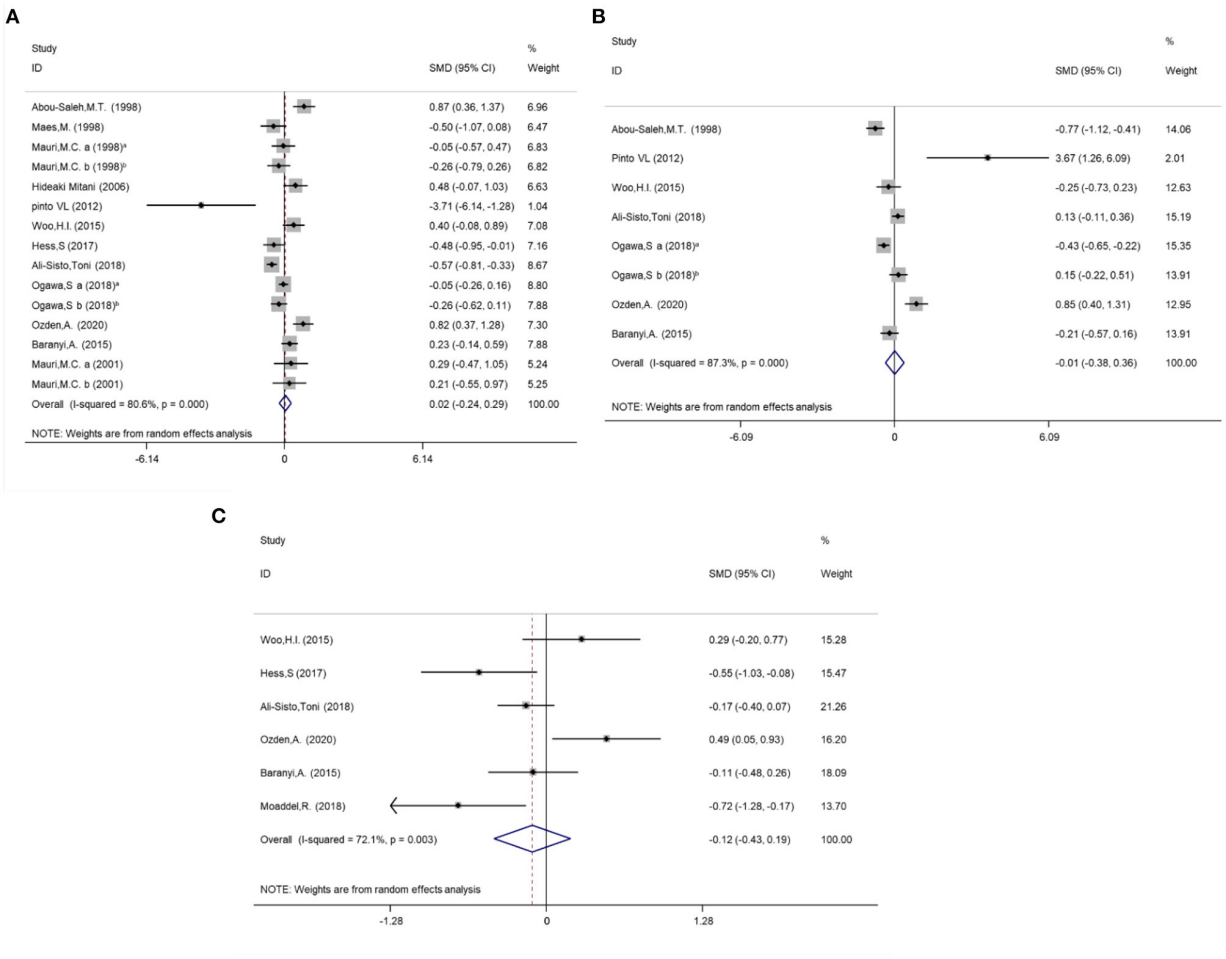
Meta-analysis for the difference of the Arginine, Ornithine and Citrulline concentrations between MDD individuals and controls by random effect analysis. **(A)** Arginine; **(B)** Ornithine. **(C)** Citrulline. Mauri et al. (54) a: sample type was plasma; Mauri et al., (54) b: sample type was platelet; Mauri et al., (55) a: sample type was plasma; Mauri et al., (55) b: sample type was platelet; Ogawa et al., (59) a: dMDD, depressed (non-remitted) individuals with major depressive disorder; Ogawa et al., (59) b: rMDD, remitted individuals.

Due to the high heterogeneity of arginine (*I*^2^ = 80.6%, *P* < 0.001), ornithine (*I*^2^ = 87.3%, *P* < 0.001) and citrulline (*I*^2^ = 72.1%, *P* = 0.003) reported in the included studies, random effect models were selected for the meta-analysis. No statistical differences in the concentrations of arginine (SMD = 0.02; 95%CI: −0.24, 0.29; *P* = 0.86), ornithine (SMD = −0.01; 95%CI: −0.38, 0.36; *P* = 0.96) and citrulline (SMD = −0.12; 95%CI: −0.43, 0.19; *P* = 0.46) were found between the subjects with MDD and HCs when serum, plasma and platelet are analyzed together. The forest plots are shown in Figure 2. Separate meta-analyses of arginine, ornithine and citrulline for serum, plasma and platelet are shown in Supplementary Figures 5–7.

### Subgroup Analysis and Meta-Regression Results

**Figure 3:**
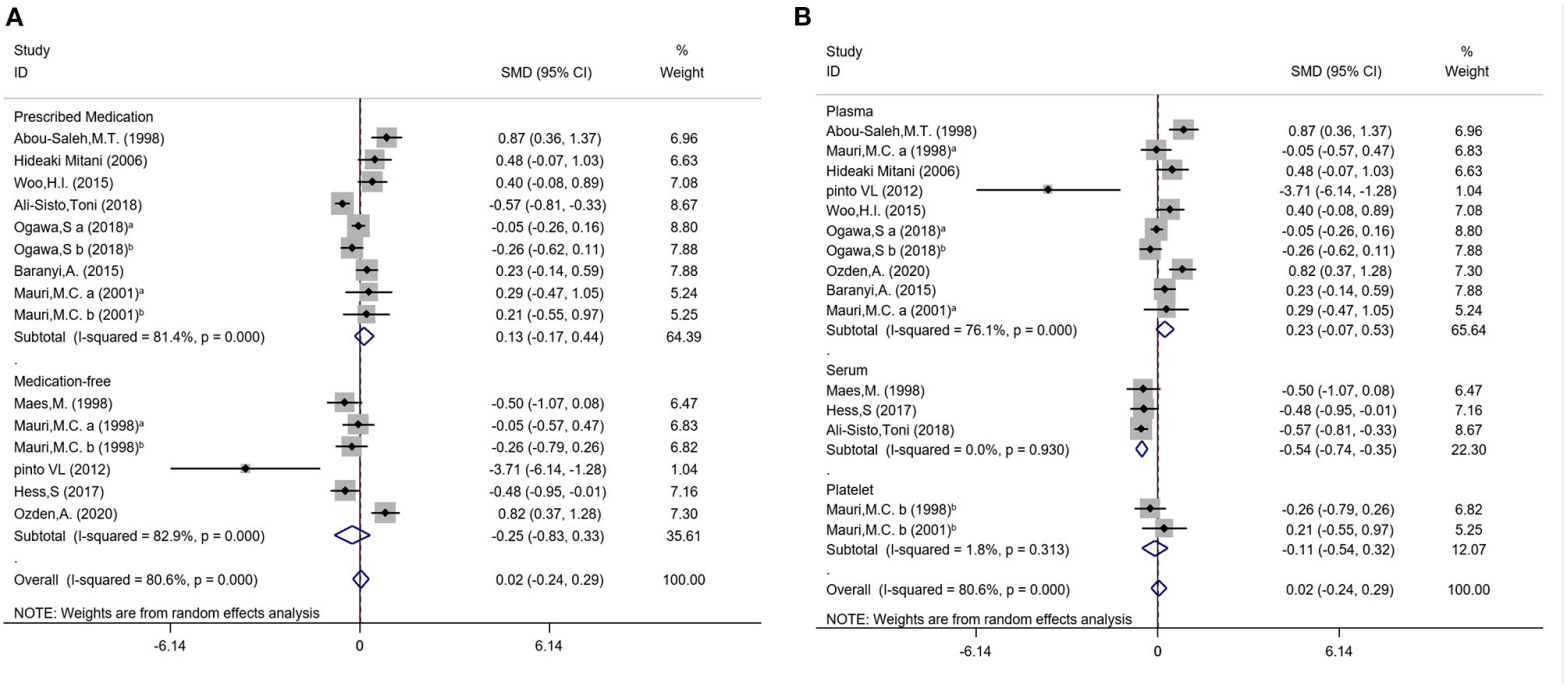
Subgroup differences of the arginine concentrations in subjects with MDD and controls. **(A)** Medication status of arginine; **(B)** Sample types of arginine.

Subgroup analysis were conducted to explore potential subgroup effects. For the subgroup analysis of arginine concentrations, the distribution trend of whether samples were prescribed medications (medication-free: SMD = −0.25; 95%CI: −0.83, 0.33; *P* = 0.39; *I*^2^ = 82.9%, *P* < 0.001; prescribed medications: SMD = 0.13; 95%CI: −0.17, 0.44; *P* = 0.39; *I*^2^ = 81.4%, *P* < 0.001) and different sample types (plasma: SMD = 0.23; 95%CI: −0.07, 0.53; *P* = 0.14; *I*^2^ = 76.1%, *P* < 0.001; serum: SMD = −0.54; *P* < 0.001; 95%CI: −0.74, −0.35; *I*^2^ = 0.0%, *P* = 0.93; platelet: SMD = −0.11; 95%CI: −0.54, 0.32; *P* = 0.62; *I*^2^ = 1.8%, *P* = 0.313) were also analyzed (Figure 3). The subgroup analysis results of medication status and sample types for ornithine and citrulline concentrations were shown in Supplementary Figure 1. We also conducted the subgroup analyses with year of publication, regional distribution, and detection methods (Supplementary Figure 2).

Meta-regression was performed on five aspects (i.e., published year, medication use status, geographic location, sample types, and detection methods) to investigate the sources of heterogeneity. None of the variables above could explain the heterogeneity of meta-analysis (all *P* > 0.05).

### Sensitivity Analysis and Publication Bias

The results of our sensitivity analysis indicated that there was no significant change by omitting a single study, indicating that the models were relatively robust (Supplementary Figure 3). The Egger's test and the Begg's test were used to evaluate the publication bias in the study (Supplementary Figure 4). The publication bias test was only performed for arginine studies since these were the only studies with a sample size greater than 10. The funnel plots of the included meta-analysis indicated that there was no publication bias of the included studies assessing the concentrations of arginine (Egger's intercept = 0.82; 95%CI = (−2.21, 3.85), *P* = 0.57 and Begg's test Z = 0.10, *P* = 0.92).

## Discussion

The main findings of the meta-analysis herein are as follows: (1) the peripheral concentrations of arginine, ornithine, and citrulline were not significantly different between patients with MDD and HCs; (2) the peripheral concentrations of arginine were lower in individuals with MDD than the HCs in the subgroups of serum samples; and (3) concurrent medication may contribute to altered peripheral concentrations of arginine, ornithine, and citrulline.

Through subgroup analysis, we found that the status of medication and sample types may contribute to the reduced heterogeneity of the sample. Although no statistically significant results were observed in the subgroup analysis of the medication status, the various trends of prescribed medication and medication-free were observed in arginine concentrations between individuals with MDD and HCs (55). Arginine concentrations reported in individuals with MDD with prescribed medication were generally higher than that of the HC population, while the arginine concentrations in individuals with MDD who were not taking medication were lower in comparison to HCs (58). Ali-Sisto et al. did not report a significant difference of arginine concentrations between responders and non-responders of antidepressants at baseline (18).

The subgroup analysis of sample types indicated that the arginine and citrulline concentrations in the serum samples were significantly lower in individuals with MDD when compared with HCs. The samples from plasma and serum represent circulating concentrations of amino acids. A previous study also illustrated that serum L-arginine concentrations do reflect intracellular L-arginine (61). Pinto et al. reported that reduced plasma L-arginine concentrations were associated with reduced L-arginine flow in platelets in a small sample comparing individuals with MDD and HCs (33).

Both the medication status and fasting status were potential confounders for the amino acid concentrations in blood samples. Psychotropic medications may have direct/indirect effects on NO metabolite concentrations in individuals with MDD. For example, medication may alter the protein-binding of amino acids, possibly leading to dysregulation in renal clearance of these metabolites. Additionally, antidepressants may cause the inhibition of related enzymes (18).

Moreover, no previous research has indicated a significant difference in amino acid concentrations between serum and EDTA-K2 anticoagulated plasma samples in individuals with MDD. A recent methodological study reported that the concentrations of amino acids were higher when compared to heparin plasma, EDTA plasma, and fluoride plasma (62). From the current meta-analysis, we cannot determine the reasons behind the observed L-arginine differences in serum vs. plasma samples between cases and controls. The current findings provide instruction from a methodological perspective on the importance of evaluating serum vs. plasma highlighting the importance of evaluating these variables separately.

To our knowledge, this is the first meta-analysis to explore the associations between arginine and related catabolites and MDD. We cannot conclude that arginine catabolism or the bioavailability of arginine has the potential to decrease or increase in individuals with MDD. Herein, our findings provide a meaningful direction for researchers engaged in the study of the metabolic mechanism of MDD. Our current findings require replication, preferrably with a large, well-characterized sample, sufficient to conduct disparate covariate analysis including buy not limited to subgrouping on the basis of medication status.

### Limitations

The findings in our study should be interpreted with caution due to the following methodological aspects affecting the outcomes of this review. Firstly, 16 studies from 13 articles were included in the current study, thus the sample sizes of overall and subgroup analysis were relatively small, and the results of subgroup analysis. Secondly, there was high heterogeneity in data comparison and data pooling. Through subgroup analysis and meta-regression analysis, the heterogeneity cannot be fully explained. Thirdly, studies that included populations categorized as “prescribed medication” may be limited in their results as some subjects may not have followed the medication regimen according to study protocol and/or included subjects that did not take any of the prescribed medication.

## Conclusion

Taken together, using meta-analytic techniques we were unable to identify compelling evidence that arginine and/or its catabolic products exhibit significant alteration in adults with MDD vs. HCs. Subgroup analysis indicated that the concentrations of arginine were lower in individuals with MDD than HCs in the subgroups of serum samples. Our findings do not exclude the possibility of a type II error due to confounding factors.

## Data Availability Statement

The original contributions presented in the study are included in the article/Supplementary Material, further inquiries can be directed to the corresponding author/s.

## Author Contributions

MF, XG, and BC conceived and designed the study. BC and LLi collected the data. LLi performed the statistical analysis. MF, RM, PD, KT, ZR, and LLui contributed to the discussion. All authors revised the paper and approved the final version of this article.

## Funding

This work was sponsored by the MOE (Ministry of Education in China) Project of Humanities and Social Sciences (21YJCZH004), Chongqing Municipal Education Commission Project of Science and Technology Innovation (KJCX2020003), and Chongqing Social Science Planning Project (2019PY57). The funding agents had no role in the design and conduct of the study; collection, management, analysis, interpretation of the data; preparation, review, or approval of the manuscript.

## Conflict of Interest

RM has received research grant support from CIHR/GACD/Chinese National Natural Research Foundation; speaker/consultation fees from Lundbeck, Janssen, Purdue, Pfizer, Otsuka, Takeda, Neurocrine, Sunovion, Bausch Health, Novo Nordisk, Kris, Sanofi, Eisai, Intra-Cellular, NewBridge Pharmaceuticals, Abbvie. RM is a CEO of Braxia Scientific Corp. KT has received personal fees from Braxia Scientific Corp. LLui is a contractor to Braxia Scientific Corp. The remaining authors declare that the research was conducted in the absence of any commercial or financial relationships that could be construed as a potential conflict of interest.

## Publisher's Note

All claims expressed in this article are solely those of the authors and do not necessarily represent those of their affiliated organizations, or those of the publisher, the editors and the reviewers. Any product that may be evaluated in this article, or claim that may be made by its manufacturer, is not guaranteed or endorsed by the publisher.
