# Supplementary material for: The Association Between Concentrations of Arginine, Ornithine, Citrulline and Major Depressive Disorder: A Meta-Analysis
Source: Front Psychiatry. 2021 Nov 18;12:686973. doi: 10.3389/fpsyt.2021.686973 (PMC8636832; doi:10.3389/fpsyt.2021.686973)
Supplement: Supplementary file 1 [file Data_Sheet_1.docx]

**Supplemental Table 1** Risk of bias assessment of included studies

| **Study** | **Selection** | | | |  | **Comparability** | |  | **Exposure/Outcome** | | | **Total score** |
| --- | --- | --- | --- | --- | --- | --- | --- | --- | --- | --- | --- | --- |
|  | **Cases definition adequate** | **Representativeness of cases** | **Selection of controls** | **Definition of controls** |  | **Age and gender** | **Additional factors** |  | **Ascertainment of outcome** | **Same evaluation method for case and controls** | **Non-response rate** |  |
| Abou-Saleh MT | * | * |  | * |  | * |  |  | * | * |  | 6 |
| Maes M | * | * |  | * |  | * | * |  | * | * |  | 7 |
| Mauri MC (1998) | * | * |  | * |  | * | * |  | * | * |  | 7 |
| Mauri MC (2001) | * | * |  | * |  | * | * |  | * | * |  | 7 |
| Mitani H | * | * |  | * |  | * |  |  | * | * |  | 6 |
| Pinto VL | * | * |  | * |  | * | * |  | * | * |  | 7 |
| Woo HI | * | * |  | * |  | * | * |  | * | * |  | 7 |
| Hess,S | * | * |  | * |  | * | * |  | * | * |  | 7 |
| Ali-Sisto T | * | * | * | * |  | * | * |  | * | * |  | 8 |
| Moaddel R | * | * |  | * |  | * | * |  | * | * |  | 7 |
| Ogawa S | * | * | * | * |  | * | * |  | * | * | * | 9 |
| Ozden A | * | * |  | * |  | * | * |  | * | * | * | 8 |
| Baranyi A | * | * |  | * |  | * | * |  | * | * | * | 8 |


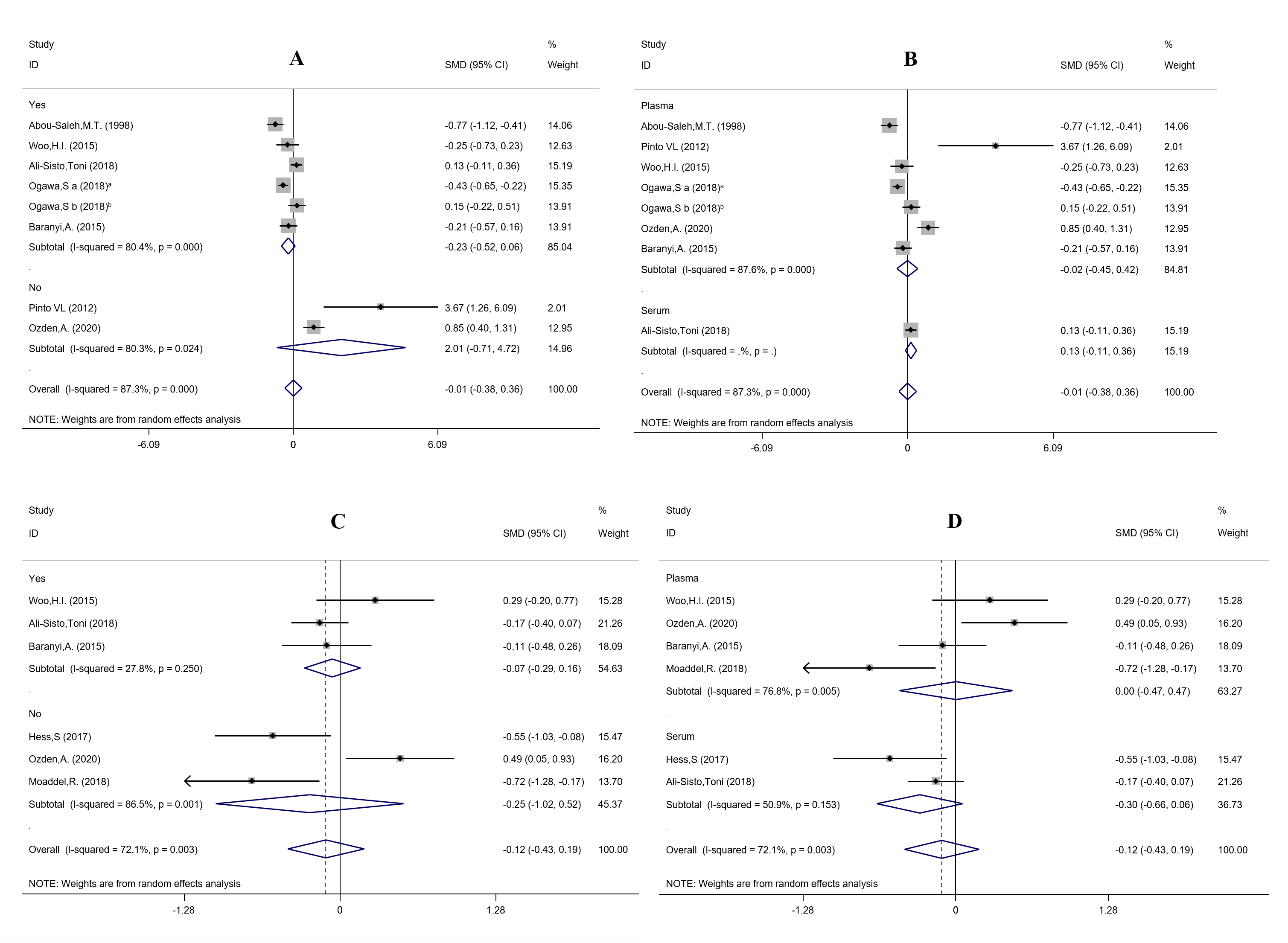


**Supplemental figure 1. Subgroup differences of the ornithine and citrulline concentrations in subjects with MDD and controls.** (A) Medication status of ornithine; (B) Sample types of ornithine; (C) Medication status of citrulline; (D) Sample types of citrulline.


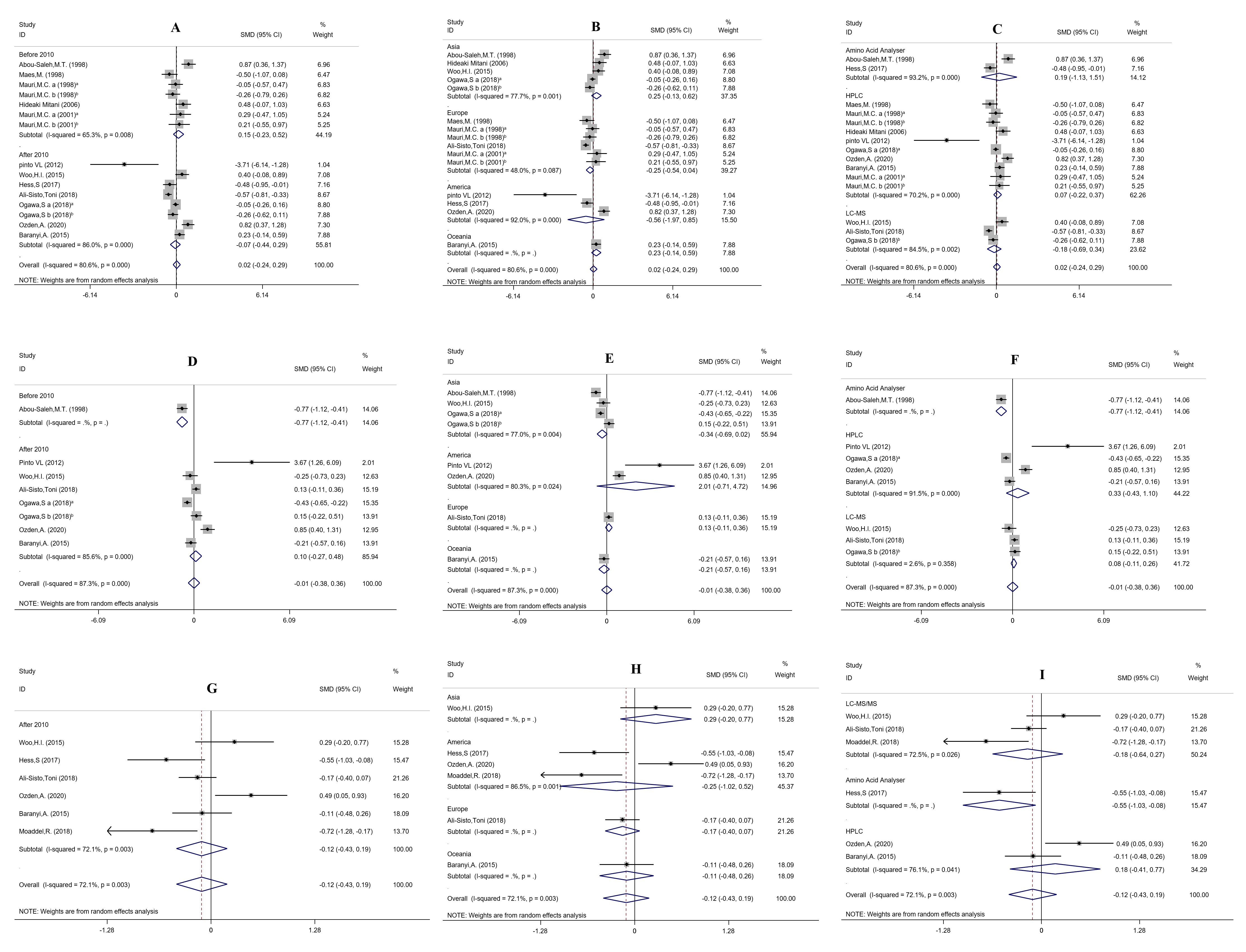


**Supplemental figure 2. Subgroup differences of the arginine, ornithine and** **citrulline concentrations in subjects with MDD individuals and controls.** (A) Published year status of arginine; (B) Regional distribution of arginine; (C) Detection method of arginine; (D) Published year status of ornithine; (E) Regional distribution of ornithine; (F) Detection method of ornithine; (G) Published year status of citrulline; (H) Regional distribution of citrulline; (I) Detection method of citrulline.

**
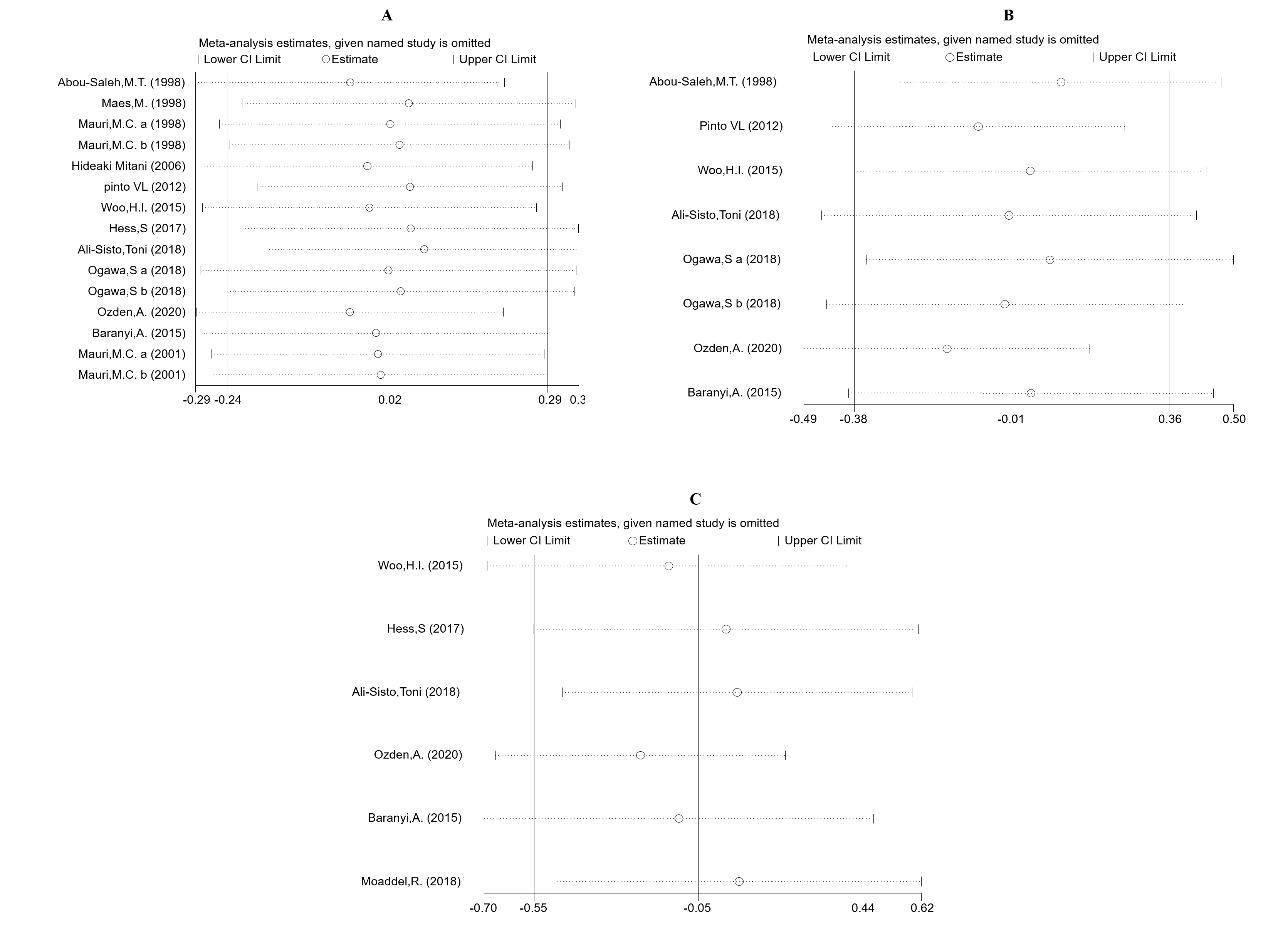
**

**Supplemental figure 3. Sensitivity analysis of the arginine, ornithine and citrulline in subjects with MDD individuals and controls.** (A) Sensitivity analysis of the arginine; (B) Sensitivity analysis of the ornithine; (C) Sensitivity analysis of the citrulline.


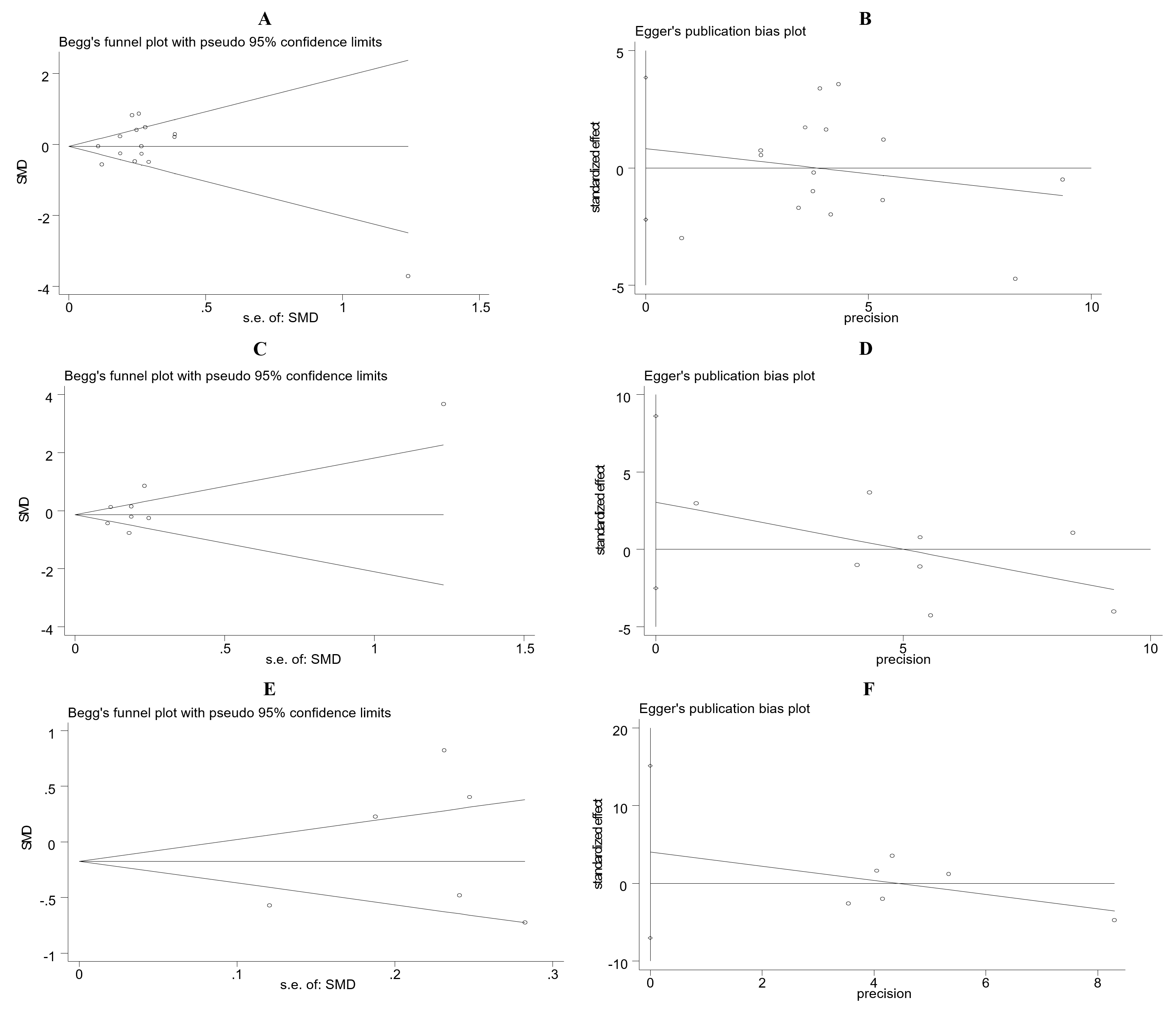


**Supplemental figure 4, Begg's and Egger's funnel plot of the estimation of publication bias.** Each point represents a separate study for the indicated association. The graph was produced using Stata 15.0. (A) and (B) Begg's and Egger's funnel plot of arginine; (C) and (D) Begg's and Egger's funnel plot of ornithine; (E) and (F) Begg's and Egger's funnel plot of citrulline.

**
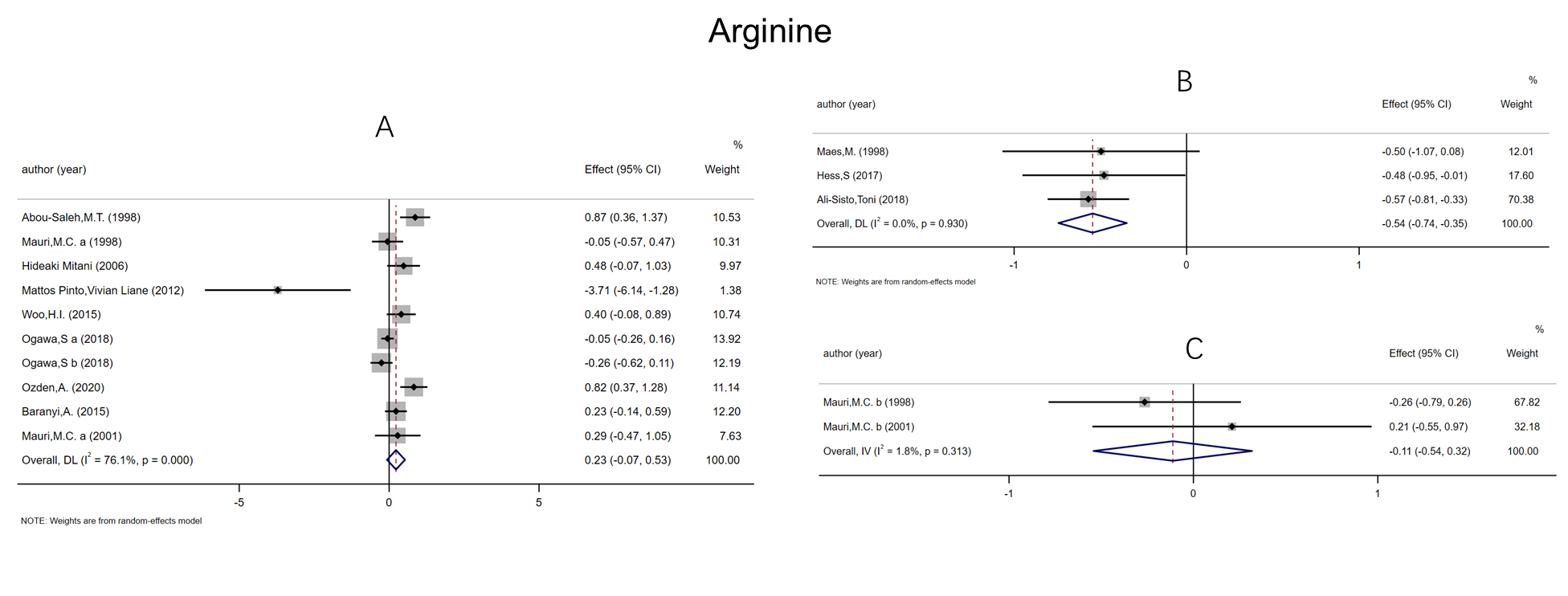
**

**Supplemental figure 5. Meta-analysis for the difference of the Arginine concentrations between MDD individuals and controls by random effect analysis.** (A) Plasma; (B) Serum; (C) Platelet.


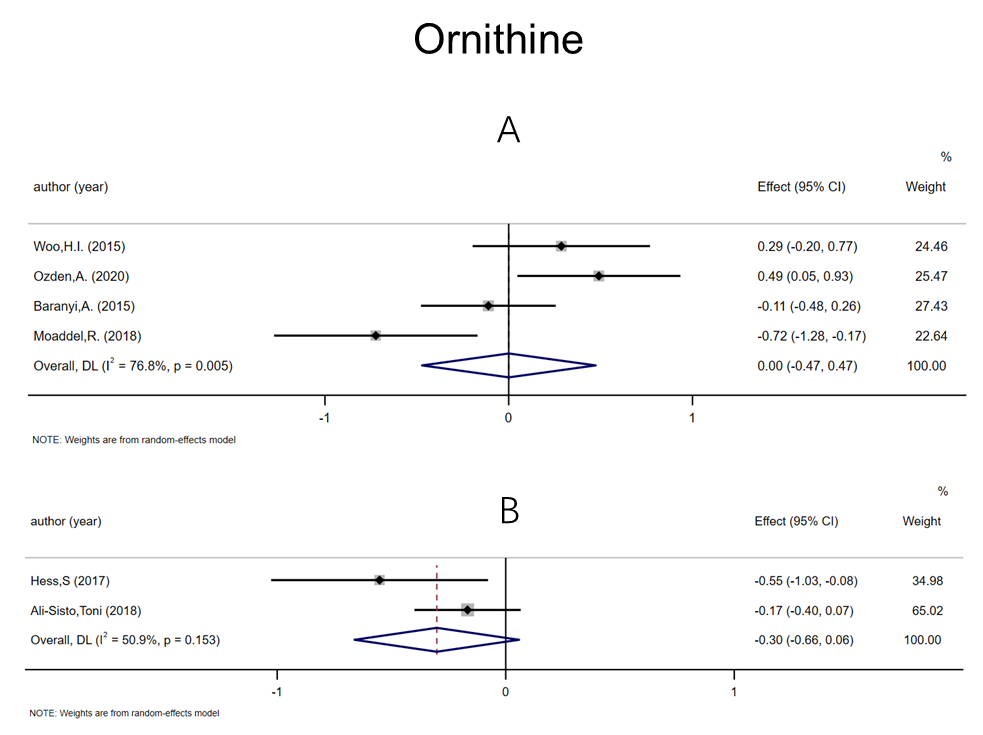


**Supplemental figure 6. Meta-analysis for the difference of the Ornithine concentrations between MDD individuals and controls by random effect analysis.** (A) Plasma; (B) Serum.


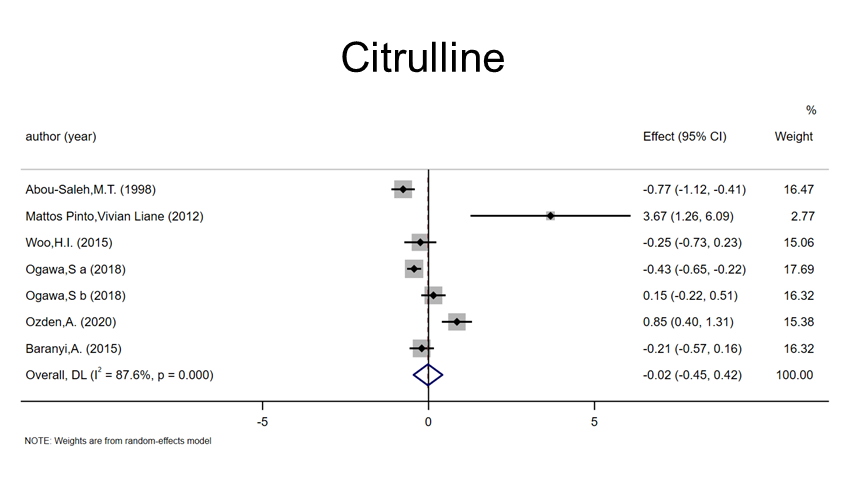


**Supplemental figure 7. Meta-analysis for the difference of the plasma citrulline concentrations between MDD individuals and controls by random effect analysis.**
